# Potential differentiation ability of gingiva originated human mesenchymal stem cell in the presence of tacrolimus

**DOI:** 10.1038/srep34910

**Published:** 2016-10-10

**Authors:** Dong-Ho Ha, Shiva Pathak, Chul Soon Yong, Jong Oh Kim, Jee-Heon Jeong, Jun-Beom Park

**Affiliations:** 1 College of Pharmacy, Yeungnam University, Gyeongsan, Gyeongsangbuk-do, 38541, Republic of Korea; 2 Department of Periodontics, College of Medicine, The Catholic University of Korea, Seoul, 06591, Republic of Korea

## Abstract

The aim of the present study is to evaluate the potential differentiation ability of gingiva originated human mesenchymal stem cell in the presence of tacrolimus. Tacrolimus-loaded poly(lactic-co-glycolic acid) microspheres were prepared using electrospraying technique. *In vitro* release study of tacrolimus-loaded poly(lactic-co-glycolic acid) microspheres was performed in phosphate-buffered saline (pH 7.4). Gingiva-derived stem cells were isolated and incubated with tacrolimus or tacrolimus-loaded microspheres. Release study of the microspheres revealed prolonged release profiles of tacrolimus without any significant initial burst release. The microsphere itself did not affect the morphology of the mesenchymal stem cells, and cell morphology was retained after incubation with microspheres loaded with tacrolimus at 1 μg/mL to 10 μg/mL. Cultures grown in the presence of microspheres loaded with tacrolimus at 1 μg/mL showed the highest mineralization. Alkaline phosphatase activity increased with an increase in incubation time. The highest expression of pSmad1/5 was achieved in the group receiving tacrolimus 0.1 μg/mL every third day, and the highest expression of osteocalcin was achieved in the group receiving 1 μg/mL every third day. Biodegradable poly(lactic-co-glycolic acid)-based microspheres loaded with tacrolimus promoted mineralization. Microspheres loaded with tacrolimus may be applied for increased osteoblastic differentiation.

Differentiation of stem cell is significant in bone tissue engineering because stem cells are considered as attractive cell sources for bone tissue engineering^1^,^2^. It was shown that the delivery of agents including drugs and growth factors can enhance the differentiation ability^3^,^4^. In this study, biodegradable microspheres were applied for the sustained delivery of drugs for the enhancement of the differentiation ability human mesenchymal stem cells.

Tacrolimus is a potent macrolide lactone immunosuppressive agent used for prophylaxis of organ rejection after transplantation and graft-versus-host disease after bone marrow transplantation in patients^5^. Tacrolimus is known to decrease the action of the immune system and this may increase the risk of viral infection^6^. Tacrolimus exerts a variety of actions on bone metabolism^4^,^7^,^8^. The osteogenic differentiation of bone marrow-derived mesenchymal stem cells is greatly promoted by the application of tacrolimus^7^. *In vivo* osteogenic capability of cultured cells in porous hydroxyapatite using a rat model showed increased bone formation with tacrolimus^8^. Similarly, our previous report showed that tacrolimus in concentrations ranging from 0.001 to 10 μg/mL did not produce statistically significant differences in the viability of stem cells derived from gingiva, but rather enhanced the osteogenic differentiation of the stem cells^4^.

A previous report showed that tacrolimus-loaded biodegradable microspheres achieved sustained release over a long period, yielding flat parallel concentration profiles for 10 days from the first day after a single subcutaneous administration in liver-transplanted rats^9^.

The aim of the present study is to evaluate the potential differentiation ability of gingiva originated human mesenchymal stem cell in the presence of tacrolimus.

## Materials and Methods

### Preparation of tacrolimus-loaded poly(lactic-co-glycolic acid) microspheres

Poly(lactic-co-glycolic acid) microspheres were fabricated using a previously reported method^10^. Tacrolimus-loaded poly(lactic-co-glycolic acid) microspheres were prepared using an electrospraying technique that involved a voltage power source from NanoNC (Seoul, Republic of Korea) supplied with a high-voltage output and a high-precision mechanical syringe pump with an adjustable flow rate. A stable jet was formed at the nozzle tip by adjusting electric voltage, flow rate, solute concentration, and collection distance between the nozzle and an aluminum sheet used as a collecting plate. Briefly, 15 mg of tacrolimus and 135 mg of poly(lactic-co-glycolic acid) were dissolved in 2 ml of methylene chloride, and the solution was sprayed for the formation of homogenous sizes of distributed microspheres.

### Characterization of tacrolimus-loaded poly(lactic-co-glycolic acid) microspheres

#### Scanning electron microscopy

To observe the surface topography of the microspheres, scanning electron microscope (S-4100, Hitachi, Japan) was used. The sample for electron microscopy was prepared by fixing the microspheres on a brass stub using double-sided adhesive tape. The microspheres were then coated with platinum for 120 seconds using Ion Sputter (E-1030, Hitachi, Japan).

#### Encapsulation efficiency and loading capacity

To determine the encapsulation efficiency and loading capacity of the microspheres, 1 mg equivalent of tacrolimus formulation was dissolved completely in acetonitrile followed by filtration using 0.5-μm filter and analysis with high-performance liquid chromatography. The composition of the mobile phase was acetonitrile and 0.1% phosphoric acid (70:30). Inertsil (4.6 × 150 mm; 5 μm) column was used for analysis. The flow rate of the mobile phase was maintained at 1 mL/minute, and a detection wavelength of 210 nm was used during the analysis. To maintain a stable flow of the mobile phase within the column, the column temperature was maintained at 40 °C. The encapsulation efficiency and loading capacity were calculated by the following mathematical relationship.


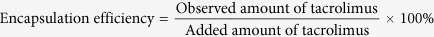



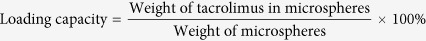


### *In vitro* release study

The *in vitro* release study of tacrolimus-loaded poly(lactic-co-glycolic acid) microspheres was performed in phosphate-buffered saline (PBS, pH 7.4; 1% tween 20) at 37 °C in a shaking incubator. Briefly, 1 mg equivalent of tacrolimus was taken in triplicate, and a suspension was made in a small volume of release medium. The suspension was then loaded in a dialysis membrane with molecular weight cut-off 3.5 kDa, and the membrane was clipped on both sides to ensure no leakage took place. The membrane was then kept in a tube containing 10 mL of release medium and incubated at the predetermined conditions. At predetermined time intervals, sampling was performed, and the whole of the release medium was replaced with an equivalent amount of fresh medium. The sampling was performed at two-day interval starting at day 1 to day 21. Two additional sampling was performed at day 25 and day 30. The release samples were analyzed by high-performance liquid chromatography analysis.

### Isolation and culture of stem cells derived from the gingiva

Gingiva-derived stem cells and cultures were obtained using a previously reported method^11^. The protocol was reviewed and approved by the Institutional Review Board of Seoul St. Mary’s Hospital, College of Medicine, The Catholic University of Korea, Seoul, Republic of Korea (KC11SISI0348), and informed consent was obtained from each patient. This study was conducted according to the Helsinki Declaration-based Ethical Principles for Medical Research Involving Human Subjects. The resected gingival tissues were de-epithelialized, minced, and digested with collagenase IV (17104019, Sigma-Aldrich Co.). The cells were incubated at 37 °C in a humidified incubator with 5% CO_2_ and 95% air. After 24 h, the non-adherent cells were washed with PBS (LB 004, Welgene, Daegu, Korea), and adherent cells were given fresh medium and fed every 2–3 days.

### Evaluation of cellular morphology

The cells were plated at a density of 2.0 × 10^3^ cells/well in 96-well plates. The cells were incubated in Minimum Essential Medium α (α-MEM) containing 15% fetal bovine serum (Gibco, Grand Island, NY, USA), 100 U/mL penicillin, and 100 μg/mL streptomycin (85856, Sigma-Aldrich Co.). Tacrolimus was dissolved in dimethyl sulfoxide (472301, Sigma-Aldrich Co.) and filter-sterilized. Equal amounts of dimethyl sulfoxide were added to each culture sample to offset the influence of this dissolving vehicle. Transwells (0.4 μm polycarbonate membrane, Corning, Inc., Corning, NY, USA) were inserted in each well. The groups consisted of (1) unloaded control group, (2) tacrolimus, 1 μg/mL (TO1), (3) tacrolimus, 10 μg/mL (TO10), (4) tacrolimus, 100 μg/mL (TO100), (5) tacrolimus, 0.1 μg/mL changed with fresh media containing tacrolimus every third day (T3/0.1), (6) tacrolimus, 1 μg/mL changed with fresh media containing tacrolimus every third day (T3/1), (7) tacrolimus, 10 μg/mL changed with fresh media containing tacrolimus every third day (T3/10), (8) unloaded microspheres (MS), (9) microspheres loaded with tacrolimus at 0.1 μg/mL (TM0.1), (10) microspheres loaded with tacrolimus at 1 μg/mL (TM1), and (11) microspheres loaded with tacrolimus at 10 μg/mL (TM10). The morphology of the cells was viewed under an inverted microscope (Leica DM IRM, Leica Microsystems, Wetzlar, Germany) on days 2 and 5.

### Determination of cell viability

The cell viability analysis was performed on days 2 and 5. The same eleven groups were used as for the cellular morphology test. WST-8 [2-(2-methoxy-4-nitrophenyl)-3-(4-nitrophenyl)-5-(2,4-disulfophenyl)-2H tetrazolium, monosodium salt] (Cell Counting Kit-8; Dojindo, Tokyo, Japan) was added to the culture, and the cells were incubated for 3 h at 37 °C. Viable cells were identified by using the Cell Counting Kit-8 (CCK-8) assay, which relies on the ability of mitochondrial dehydrogenases to oxidize WST-8 to a formazan product. The spectrophotometric absorbance at 450 nm was measured using a microplate reader (BioTek, Winooski, VT, USA). The tests were performed in triplicate.

### Alizarin Red S staining

Cell cultures grown with osteogenic media were obtained on days 7 and 14, washed twice with PBS (Welgene), fixed with 70% ethanol, and rinsed twice with deionized water. Cultures were stained with Alizarin Red S for 30 minutes at room temperature. To remove non-specifically bound stain, cultures were washed three times with deionized water and once with PBS for 15 minutes at ambient temperature. Bound dye was solubilized in 10 mM sodium phosphate containing 10% cetylpyridinium chloride and quantified spectrophotometrically at 560 nm.

### Alkaline phosphatase activity assays

Cell cultures grown with osteogenic media were obtained on days 5 and 7. Cells were detached using trypsin (Gibco) and washed with PBS (Welgene). Alkaline phosphatase activity assays were done with a commercially available kit (K412-500, BioVision, Inc., Milpitas, CA, USA). The cells were resuspended in assay buffer, sonicated, and then centrifuged to remove insoluble material. Supernatant was mixed with p-nitrophenylphosphate substrate and incubated at 25 °C for 60 minutes. The optical density of resultant p-nitrophenol at 405 nm was determined spectrophotometrically.

### Western blot analysis

Stem cells were washed two times with ice-cold PBS and solubilized in lysis buffer at day 3 and day 5. The lysates were centrifuged at 15,000 rpm for 10 minutes at 4 °C. These samples were then separated by sodium dodecyl sulfate polyacrylamide gel electrophoresis, transferred to polyvinylidene difluoride membranes (Immun-Blot^®^, Bio-Rad, Hercules, CA, USA), and immunoblotted with the corresponding antibodies and enhanced chemiluminescent detection kits. Primary antibodies against pSmad1/5, osteocalcin, β-actin and secondary antibodies were purchased from Cell Signaling Technology, Inc. (Danvers, MA, USA), Thermo Pierce^TM^ (Rockford, IL, USA), and Santa Cruz Biotechnology (Santa Cruz, CA, USA). Quantitative analysis of the protein expressions of pSmad1/5, osteocalcin, and β-actin was conducted with image processing and analyzing software (ImageJ, National Institutes of Health, Bethesda MD, USA). The groups consisted of (1) unloaded control group, (2) tacrolimus, 0.1 μg/mL changed with fresh media containing tacrolimus every third day (T3/0.1), (3) tacrolimus, 1 μg/mL changed with fresh media containing tacrolimus every third day (T3/1), (4) tacrolimus, 10 μg/mL changed with fresh media containing tacrolimus every third day (T3/10), (5) unloaded microspheres (MS), (6) microspheres loaded with tacrolimus at 0.1 μg/mL (TM0.1), (7) microspheres loaded with tacrolimus at 1 μg/mL (TM1), and (8) microspheres loaded with tacrolimus at 10 μg/mL (TM10).

### Statistical analysis

The data are presented as means ± standard deviations of the experiments. One-way analysis of variance (ANOVA) with post hoc test was performed to determine the differences between groups using a commercially available program (SPSS 12 for Windows, SPSS Inc., Chicago, IL, USA). The level of significance was 0.05.

## Results

### Characterization of tacrolimus-loaded poly(lactic-co-glycolic acid) microspheres

#### Scanning electron microscopy

**Figure 1:**
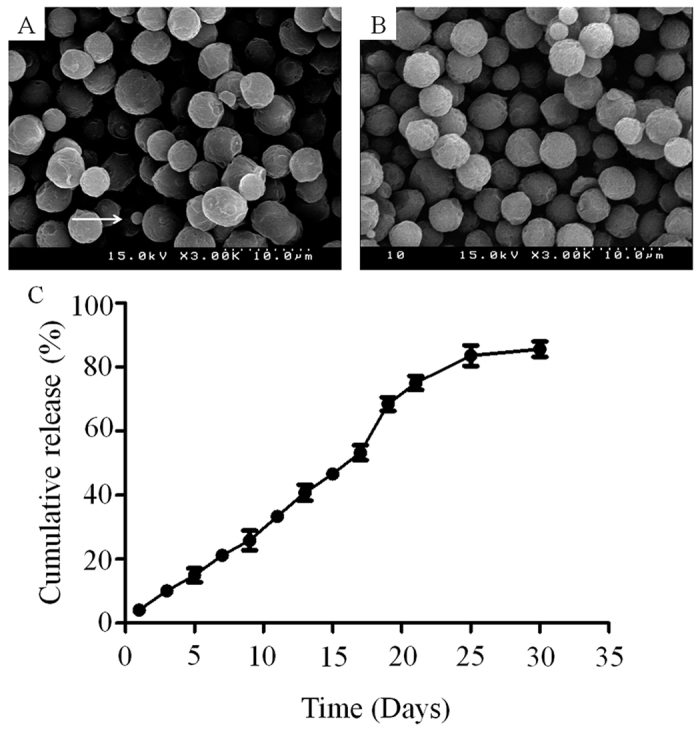
(**A**) Characterization of blank microsphere using scanning electron microscope (Arrow indicates small particle due to the Coulomb fission which occurred during the evaporation of organic solvent). (**B**) Characterization of microsphere loaded with tacrolimus using scanning electron microscope under optimized conditions. (**C**) Evaluation of the release profile.

Figure 1 shows the scanning electron microscopy of tacrolimus-loaded poly(lactic-co-glycolic acid) microspheres. As revealed from the microscopy, homogenously size-distributed, drug-loaded microspheres were obtained by electrospraying. The diameter of microspheres ranged from 3 to 6 μm (Fig. 1A,B). Because of the Coulomb fission that occurs during the evaporation of organic solvent, a few small particles were also observed (arrow).

#### Encapsulation efficiency and loading capacity

The encapsulation efficiency and loading capacity were 90.77 ± 0.95% and 9.41 ± 0.41%, respectively. Due to the sufficiently hydrophobic nature of tacrolimus, it was well incorporated inside the PLGA microspheres, leading to good encapsulation efficiency.

### *In vitro* release study

The release study of the microspheres revealed prolonged release profile of tacrolimus, extending to more than 25 days without a significant initial burst release (Fig. 1C). A high degree of correlation (r^2^ = 0.9728) to zero order equation (Q_o_-Q_t_ = K_o_t, where ‘Q’ is the amount of drug dissolved in time ‘t’, ‘Q_o_’ is the initial amount of drug in the solution, and ‘K_o_’ is the zero order rate constant) was found, indicating that the drug release is independent of the concentration of loaded drug in the microspheres. Moreover, the release profile also showed good correlation (r^2^ = 0.9897) with Korsmeyer-Peppas model (M_t_/M_∞_ = K.t^n^, where ‘M_t_/M_∞_’ is the fraction of drug released after time ‘t’, ‘K’ is the release constant and ‘n’ is the release exponent, which characterizes the different release mechanisms), indicating that diffusion was the primary mechanism involved in the drug release from the formulation. Also, the n value in Korsmeyer-Peppas model suggested that the mechanism of drug release is related to drug diffusion as well as polymer degradation.

### Evaluation of cell morphology

**Figure 2:**
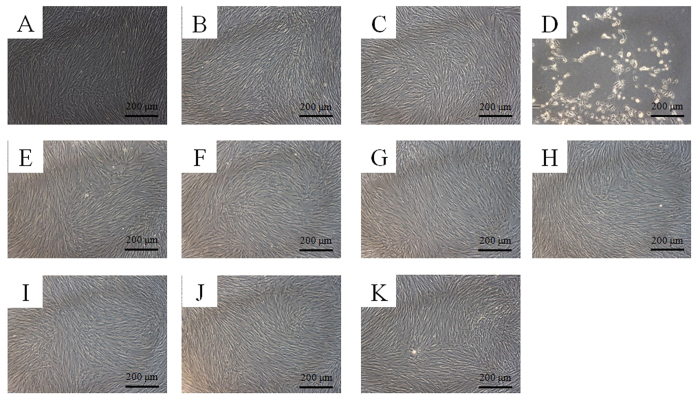
Morphology of the stem cells on day 2. (**A**) Unloaded control group. (**B**) Tacrolimus, 1 μg/Ml (TO1). (**C**) Tacrolimus, 10 μg/mL (TO10). (**D**) Tacrolimus, 100 μg/mL (TO100). (**E**) Tacrolimus (0.1 μg/mL) changed with fresh media containing tacrolimus every third day (T3/0.1). (**F**) Tacrolimus (1 μg/mL) changed with fresh media containing tacrolimus every third day (T3/1). (**G**) Tacrolimus (10 μg/mL) changed with fresh media containing tacrolimus every third day (T3/10). (**H**) Unloaded microspheres (MS). (**I**) Microspheres loaded with tacrolimus at 0.1 μg/mL (TM0.1). (**J**) Microspheres loaded with tacrolimus at 1 μg/mL (TM1). (**K**) Microspheres loaded with tacrolimus at 10 μg/mL (TM10).

The control group showed normal fibroblast morphology on day 2 (Fig. 2A). The shapes of the cells in 1 μg/mL (Fig. 2B) and 10 μg/mL (Fig. 2C) tacrolimus; T3/0.1 group (Fig. 2E), T3/1 group (Fig. 2F), T3/10 group (Fig. 2G); MS group (Fig. 2H); and TM0.1 group (Fig. 2I), TM1 group (Fig. 2J), and TM10 group (Fig. 2K) were similar to those of the control group. However, the 100 μg/ml group showed significant differences compared to the control group (Fig. 2D). The shapes of the cells in the 100 μg/mL group were rounder, and fewer cells were present.

**Figure 3:**
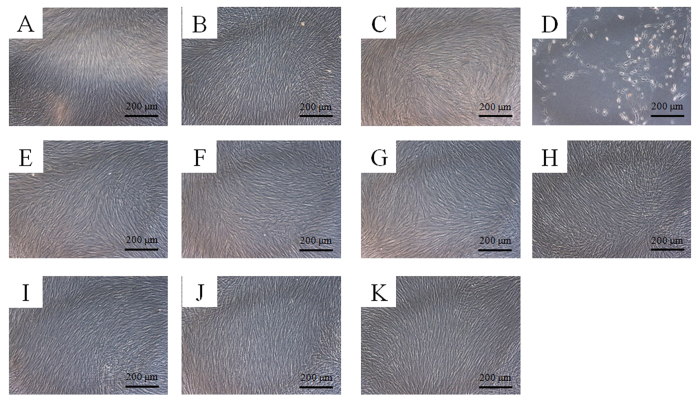
Morphology of the stem cells on day 5. (**A**) Unloaded control group. (**B**) Tacrolimus, 1 μg/mL (TO1). (**C**) Tacrolimus, 10 μg/mL (TO10). (**D**) Tacrolimus, 100 μg/mL (TO100). (**E**) Tacrolimus (0.1 μg/mL) changed with fresh media containing tacrolimus every third day (T3/0.1). (**F**) Tacrolimus (1 μg/mL) changed with fresh media containing tacrolimus every third day (T3/1). (**G**) Tacrolimus (10 μg/mL) changed with fresh media containing tacrolimus every third day (T3/10). (**H**) Unloaded microspheres (MS). (**I**) Microspheres loaded with tacrolimus at 0.1 μg/mL (TM0.1). (**J**) Microspheres loaded with tacrolimus at 1 μg/mL (TM1). (**K**) Microspheres loaded with tacrolimus at 10 μg/mL (TM10).

The morphology of the cells on day 5 is shown in Fig. 3. The shapes of the cells in the tested groups were similar to the control group except for the 100 μg/mL group.

### Cellular viability

**Figure 4:**
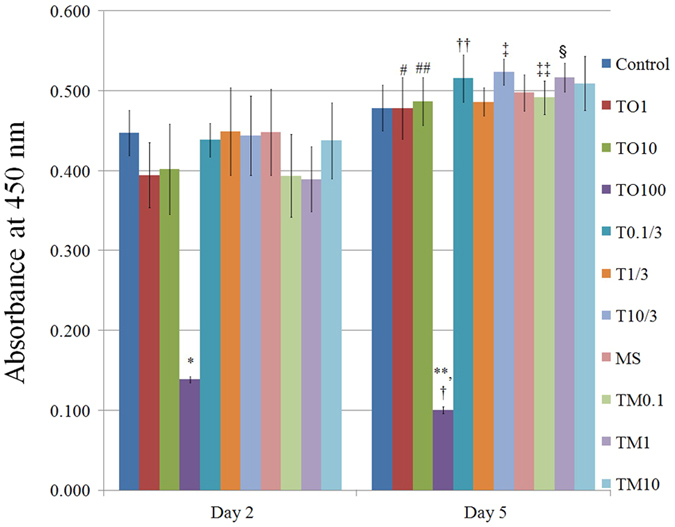
Cellular viability using CCK-8 assay at days 2 and 5. *Statistically significant differences noted versus control on day 2. **Statistically significant differences noted when compared with the control on day 5. ^#^Significant differences were noted versus TO1 group on day 2. ^##^Significant differences were noted versus TO10 group on day 2. ^†^Significant differences were noted versus TO100 group on day 2. ^††^Significant differences were noted versus T 0.1/3 group on day 2. ^‡^Significant differences were noted versus T 10/3 group on day 2. ^‡‡^Significant differences were noted versus TM 0.1 group on day 2. ^§^Significant differences were noted versus TM 1 group on day 2.

The CCK-8 results on days 2 and 5 are shown in Fig. 4. Compared to the control, growth in the presence of tacrolimus at 100 μg/mL resulted in decreases in the CCK-8 values on days 2 and 5, respectively (*P* *<* 0.05). All groups except the unloaded control, the 100 μg/mL group, and microspheres loaded with tacrolimus at 10 μg/mL showed statistically significant increases in cell proliferation over time.

### Mineralization assay

**Figure 5:**
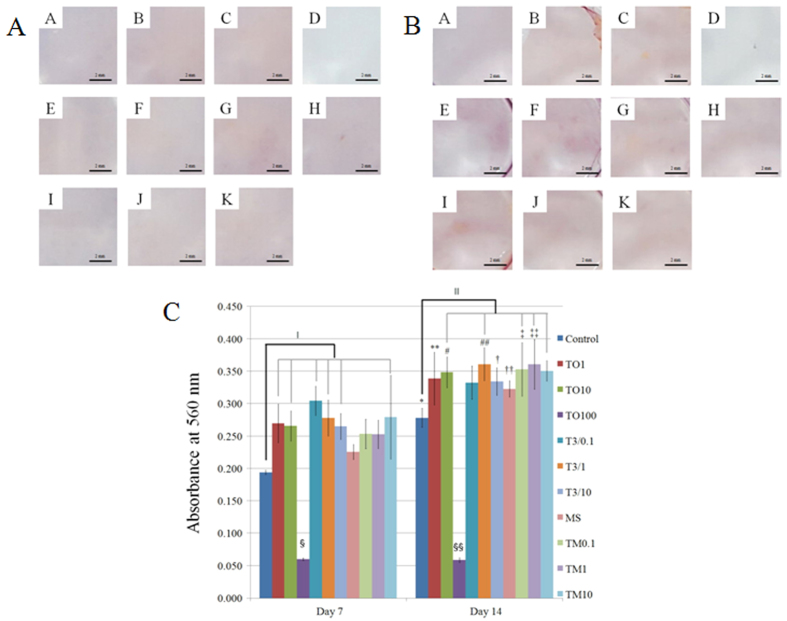
(**A**) Results of Alizarin Red S staining on day 7. (A) Unloaded control group. (B) Tacrolimus, 1 μg/mL (TO1). (C) Tacrolimus, 10 μg/mL (TO10). (D) Tacrolimus, 100 μg/mL (TO100). (E) Tacrolimus (0.1 μg/mL) changed with fresh media containing tacrolimus every third day (T3/0.1). (F) Tacrolimus (1 μg/mL) changed with fresh media containing tacrolimus every third day (T3/1). (G) Tacrolimus (10 μg/mL) changed with fresh media containing tacrolimus every third day (T3/10). (H) Unloaded microspheres (MS). (I) Microspheres loaded with tacrolimus at 0.1 μg/mL (TM0.1). (J) Microspheres loaded with tacrolimus at 1 μg/mL (TM1). (K) Microspheres loaded with tacrolimus at 10 μg/mL (TM10). (**B**) Results of Alizarin Red S staining on day 14. (A) Unloaded control group. (B) Tacrolimus, 1 μg/mL (TO1). (C) Tacrolimus, 10 μg/mL (TO10). (D) Tacrolimus, 100 μg/mL (TO100). (E) Tacrolimus (0.1 μg/mL) changed with fresh media containing tacrolimus every third day (T3/0.1). (F) Tacrolimus (1 μg/mL) changed with fresh media containing tacrolimus every third day (T3/1). (G) Tacrolimus (10 μg/mL) changed with fresh media containing tacrolimus every third day (T3/10). (H) Unloaded microspheres MS). (I) Microspheres loaded with tacrolimus at 0.1 μg/mL (TM0.1). (J) Microspheres loaded with tacrolimus at 1 μg/mL (TM1). (K) Microspheres loaded with tacrolimus at 10 μg/mL (TM10). (**C**) Quantitative results of mineralization assay on days 7 and 14. *Statistically significant differences noted versus control on day 7. **Statistically significant differences noted when compared with TO 1 on day 7. ^#^Significant differences were noted versus TO 10 group on day 7. ^##^Significant differences were noted versus T3/1 group on day 7. ^†^Significant differences were noted versus T3/10 group on day 7. ^††^Significant differences were noted versus MS group on day 7. ^‡^Significant differences were noted versusTM0.1 group on day 7. ^‡‡^Significant differences were noted versus TM1 group on day 7. ^‖^Significant differences were noted versus control on day 7. ^‖‖^Significant differences were noted versus control on day 14.

Mineralized extracellular deposits were minimally observed after Alizarin Red S staining on days 7 and 14 (Fig. 5A,B). Increase of mineralized deposits was noted on day 14 when compared with day 7. The quantitative results regarding bound dye on days 7 and 14 are shown in Fig. 5C.

Cultures grown in the presence of 1 μg/mL and 10 μg/mL tacrolimus; T3/0.1 group, T3/1 group and T3/10 group; and TM10 group showed statistically significant increases of mineralized deposits compared with the control on day 7.

The results for day 14 showed that treatment with tacrolimus generally increased deposits compared with those on day 7 in each group. Cultures grown in the presence of 10 μg/mL tacrolimus, groups of 1 μg/mL changed with fresh media containing tacrolimus every third day, and microspheres loaded with tacrolimus at 0.1, 1, and 10 μg/mL showed statistically significant increases compared with the control on day 14.

### Alkaline phosphatase activity assays

**Figure 6:**
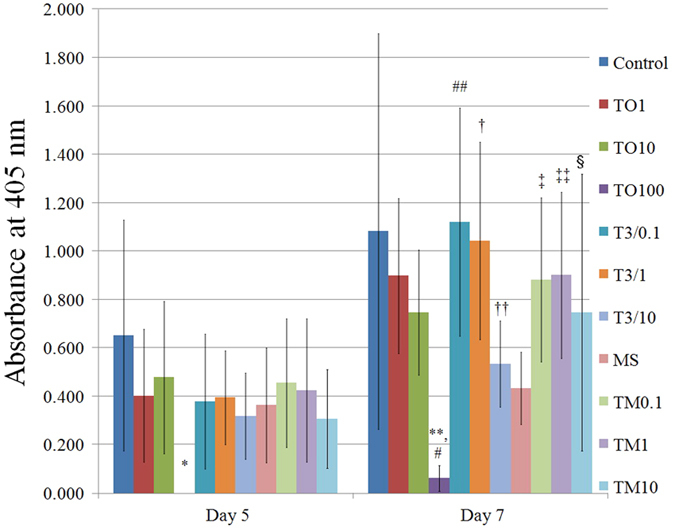
Alkaline phosphatase activity on days 5 and 7. ^*^Statistically significant differences noted versus control on day 5. ^**^Statistically significant differences noted versus control on day 7. ^#^Significant differences were noted versus TO100 group on day 5. ^##^Significant differences were noted versus T3/0.1 group on day 5. ^†^Significant differences were noted versus T3/1 group on day 5. ^††^Significant differences were noted versus T3/10 group on day 5. ^‡^Significant differences were noted versus TM 0.1 group on day 5. ^‡‡^Significant differences were noted versus TM1 group on day 5. ^§^Significant differences were noted versus TM10 group on day 5.

For mesenchymal stem cells treated with tacrolimus, the alkaline phosphatase activity was increased between 5 and 7 days with statistically significant differences achieved with 100 μg/mL tacrolimus; T3/0.1 group, T3/1 group and T3/10 group; TM0.1 group, TM1 group, and TM10 group (Fig. 6).

The differences in the alkaline phosphatase activity between the groups did not reach statistical significance. However, a statistically significant decrease was observed only between the control group and 100 μg/mL tacrolimus group on days 5 and 7 (*P* < 0.05).

### Western blot

**Figure 7:**
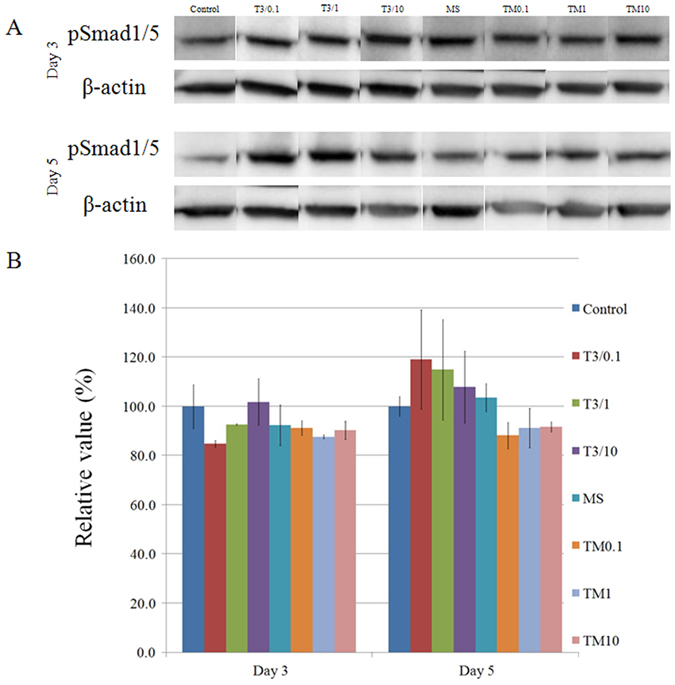
(**A**) Western blot analysis to detect the protein expressions of pSmad1/5 and β-actin on day 3 and day 5. The gels have been under the same experimental conditions. The full-length blots are included in the supplementary information. (**B**) Quantitative analysis of the protein expressions of pSmad1/5 after normalization with β-actin levels by densitometry.

Western blot analysis was performed to detect protein expression of pSmad1/5 and osteocalcin following treatment with tacrolimus. Normalization of the protein expressions of pSmad1/5 on day 3 revealed that (2) T3/0.1 group, (3) T3/1 group, (4) T3/10 group, (5) MS group, (6) TM0.1 group, (7) T3/1 group, and (8) T3/10 group yielded 84.8 ± 20.1%, 92.7 ± 20.4%, 101.8 ± 14.6%, 92.3 ± 5.8%, 91.2 ± 5.3%, 87.6 ± 8.0%, and 90.2 ± 2.1% pSmad1/5 expression, respectively, when (1) unloaded control group was considered 100% (100.0 ± 8.9%) (Fig. 7A,B).

Normalization of the protein expressions on day 5 revealed that (2) T3/0.1 group, (3) T3/1 group, (4) T3/10 group, (5) MS group, (6) TM0.1 group, (7) T3/1 group, and (8) T3/10 group yielded 119.1 ± 20.1%, 115.0 ± 20.4%, 107.8 ± 14.6%, 103.6 ± 5.8%, 88.1 ± 5.3%, 91.2 ± 8.0%, and 91.7 ± 2.1% pSmad1/5 expression, respectively, when (1) unloaded control group was considered 100% (100.0 ± 4.0%) (Fig. 7A,B).

**Figure 8:**
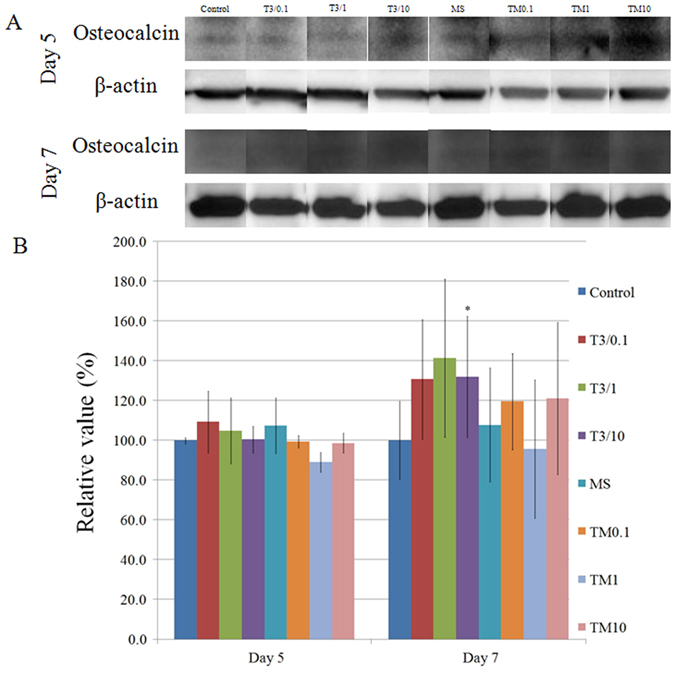
(**A**) Western blot analysis to detect the protein expressions of osteocalcin and β-actin on day 5 and day 7. The gels have been under the same experimental conditions. The full-length blots are included in the supplementary information. (**B**) Quantitative analysis of the protein expressions of osteocalcin after normalization with β-actin levels by densitometry *Statistically significant differences noted versus T3/10 on day 5.

Normalization of the protein expressions of osteocalcin on day 5 revealed (2) T3/0.1 group, (3) T3/1 group, (4) T3/10 group, (5) MS group, (6) TM0.1 group, (7) T3/1 group, and (8) T3/10 group yielded 109.4 ± 15.6%, 105.0 ± 16.6%, 100.5 ± 6.7%, 107.3 ± 14.0%, 99.4 ± 3.1%, 89.1 ± 4.9%, and 98.7 ± 5.0% osteocalcin expression, respectively, when (1) unloaded control group was considered 100% (100.0 ± 1.6%) (Fig. 8A,B).

Normalization of the protein expressions of osteocalcin on day 7 revealed that (2) T3/0.1 group, (3) T3/1 group, (4) T3/10 group, (5) MS group, (6) TM0.1 group, (7) T3/1 group, and (8) T3/10 group yielded 130.9 ± 30.1%, 141.5 ± 39.7%, 132.0 ± 30.6%, 107.8 ± 28.6%, 119.6 ± 24.1%, 95.8 ± 34.9%, and 121.1 ± 38.3% osteocalcin expression, respectively, when (1) unloaded control group was considered 100% (100.0 ± 19.8%). The increase of osteocalcin expression at the (4) tacrolimus of 10 μg/mL changed with fresh media containing tacrolimus every third day reached a statistically significant level (*P* < 0.05) (Fig. 8A,B).

## Discussion

This report discusses the effects of biodegradable poly(lactic-co-glycolic acid)-based microspheres loaded with tacrolimus on the proliferation and differentiation of mesenchymal stem cells derived from the gingiva.

This study used an electrospray technique with a single-nozzle electrospraying machine. A previous study used electrospraying to prepare poly(lactic-co-glycolic acid) microparticles and further encapsulate the drug, and electrospraying was concluded to be a cost-effective, single-step process for the preparation of polymeric microparticles^12^. Scanning electron microscopy revealed a uniform round shape, and loading of tacrolimus did not interfere with microsphere formation. The size and charge of the particles could be controlled by regulating the polymer solution flow rate and electric voltage^13^. This study showed that microsphere formation led to prolonged release of the drug without significant initial burst. Similarly, previous research reported electrosprayed poly(lactic-co-glycolic acid) microdevices for sustained drug delivery for over three weeks^14^. The release profile could be controlled by the drug:polymer ratio, and previous results showed that the drug:polymer ratio of 1:10 had slower release profiles than those with a 1:5 ratio^15^.

Cytotoxicity evaluation of the microsphere itself did not affect the morphology of the mesenchymal stem cells. Moreover, cell morphology was retained after incubation with microspheres loaded with tacrolimus at final concentration of 1 μg/mL to 10 μg/mL. However, higher doses of tacrolimus (100 μg/mL) produced rounder shapes with fewer cells. Cellular viability was evaluated using CCK-8 assay, which is based on mitochondrial enzyme reduction of the water-soluble tetrazolium salt-8-(2(2-methoxy-4-nitrophenyl)-3-(4-nitrophenyl)-5-(2,4-disulphophenyl)-2H-tetrazolium monosodium salt) and quantification of any generated water-soluble formazan^16^. Cellular viability can be evaluated using various methods^17^,^18^,^19^. The dye exclusion test is based on the principle that live cells possess intact cell membranes that exclude certain dyes, such as trypan blue^17^. The protein assay is an indirect measurement of cell viability because it measures the protein content of viable cells that are left after washing the treated plates^18^,^19^. Thus, CCK-8 assay can be regarded as a more sensitive assay because it measures cell viability through the determination of mitochondrial dehydrogenase activity. It should be noted that the use of higher doses of tacrolimus was previously shown to yield a negative effect on cell viability^20^, and our study proved that tacrolimus at 100 μg/mL decreased cell viability on days 2 and 5.

Following the period of matrix maturation, nodule cells begin to mineralize the extracellular matrix^21^. Alizarin Red S staining was used to evaluate the presence of calcium deposits, and cetylpyridinium chloride was applied for the quantitative analysis^22^. A previous report showed that tacrolimus at 0.04 μg/mL and 0.4 μg/mL enhanced osteoblastic differentiation of mesenchymal stem cells^20^. Our previous research showed that tacrolimus showed the highest mineralized nodule formation at 0.001, 0.01, and 1 μg/mL^4^. In this report, cultures grown in the presence of microspheres loaded with tacrolimus at 1 μg/mL showed the highest mineralization.

Alkaline phosphatase activity is considered an early marker of osteogenic differentiation^23^. Alkaline phosphatase activities increased between 5 and 7 days, but there were no significant differences noted except for the 100 μg/mL group. However, higher alkaline phosphatase activity was previously noted in the allografts with tacrolimus^24^. The differences in the responses may be explained by the types and stages of the cells, the culturing time period, and the system^25^.

Western blot analysis was performed to detect protein expression of pSmad1/5 and osteocalcin following treatment with tacrolimus to provide information of additional possible mechanisms. Tacrolimus may influence osteoblast differentiation through bone morphogenetic protein signaling^26^. Highest expression of pSmad1/5 was achieved in the tacrolimus (0.1 μg/mL) group changed with fresh media containing tacrolimus every third day. A previous report showed that co-stimulation with tacrolimus at 1.0 μg/mL and bone morphogenetic protein-9 at 100 ng/mL induced remarkable osteoblastic differentiation^27^. Osteocalcin is reported to be an osteoblast-specific gene expressed by fully differentiated osteoblasts^28^. Highest expression of osteocalcin was achieved in the tacrolimus (1 μg/mL) group changed with fresh media containing tacrolimus every third day, but it did not reach statistical significance.

Based on these findings, biodegradable poly(lactic-co-glycolic acid)-based microspheres loaded with tacrolimus produced prolonged release profiles with increased mineralization. Microspheres loaded with tacrolimus may be applied for increased osteoblastic differentiation.

## Additional Information

**How to cite this article**: Ha, D.-H. *et al*. Potential differentiation ability of gingiva originated human mesenchymal stem cell in the presence of tacrolimus. *Sci. Rep.* **6**, 34910; doi: 10.1038/srep34910 (2016).

## Supplementary Material

Supplementary Information

Supplementary Fig. 1

Supplementary Fig. 2
