# Supplementary material for: Potential differentiation ability of gingiva originated human mesenchymal stem cell in the presence of tacrolimus
Source: Sci Rep. 2016 Oct 10;6:34910. doi: 10.1038/srep34910 (PMC5056516; doi:10.1038/srep34910)
Supplement: Supplementary Information [file srep34910-s1.doc]

**Potential differentiation ability of gingiva originated human mesenchymal stem cell in the presence of tacrolimus**

Dong-Ho Ha^1*^, Shiva Pathak^1*^, Chul Soon Yong PhD^1^, Jong Oh Kim PhD^1^, Jee-Heon Jeong PhD^1^, Jun-Beom Park, DDS, MSD, PhD^2^

^1^College of Pharmacy, Yeungnam University, Gyeongsan, Gyeongsangbuk-do, 38541, Republic of Korea

^2^Department of Periodontics, College of Medicine, The Catholic University of Korea, Seoul, 06591, Republic of Korea

**^*^**These authors contributed equally as first authors.

**Corresponding author**

Jee-Heon Jeong, PhD

College of Pharmacy, Yeungnam University, 280 Daehak-Ro, Gyeongsan-si, Gyeongsangbuk-do, 38541, Republic of Korea

Phone: + 82-53-810-2822

Fax: +82-53-810-4654

E-mail: jeeheon@yu.ac.kr

Jun-Beom Park, DDS, MSD, PhD

Department of Periodontics, Seoul St Mary’s Hospital, College of Medicine, The Catholic University of Korea

222 Banpo-daero, Seocho-gu, Seoul, 06591, Republic of Korea

Phone: +82-10-4325-2651

Fax: +82-2-537-2374

E-mail: [jbassoonis@yahoo.co.kr](mailto:jbassoonis@yahoo.co.kr)

**Running title:** Microspheres with tacrolimus on stem cells

Supplementary Fig. 1.

A. Western blot of pSmad1/5 on day 3.

B. Western blot of β-actin on day 3.

C. Western blot of pSmad1/5 on day 5.

D. Western blot of β-actin on day 5.

Supplementary Fig. 2.

A. Western blot of osteocalcin1/5 on day 5.

B. Western blot of β-actin on day 5.

C. Western blot of osteocalcin on day 7.

D. Western blot of β-actin on day 7.

Supplementary Fig. 1.

***
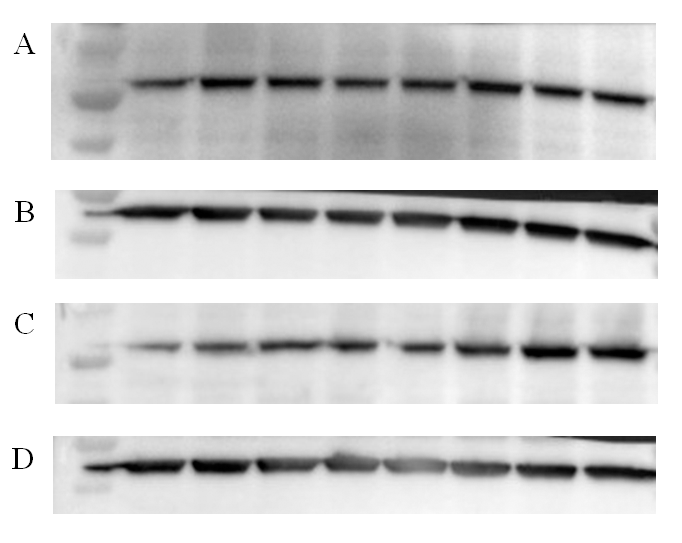
***

Supplementary Fig. 2.

***
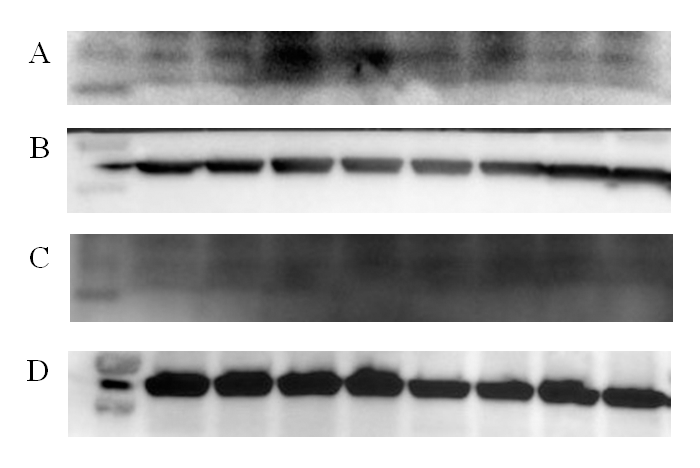
***
