# Supplementary figures and images for: Potential differentiation ability of gingiva originated human mesenchymal stem cell in the presence of tacrolimus
Source: Sci Rep. 2016 Oct 10;6:34910. doi: 10.1038/srep34910 (PMC5056516; doi:10.1038/srep34910)

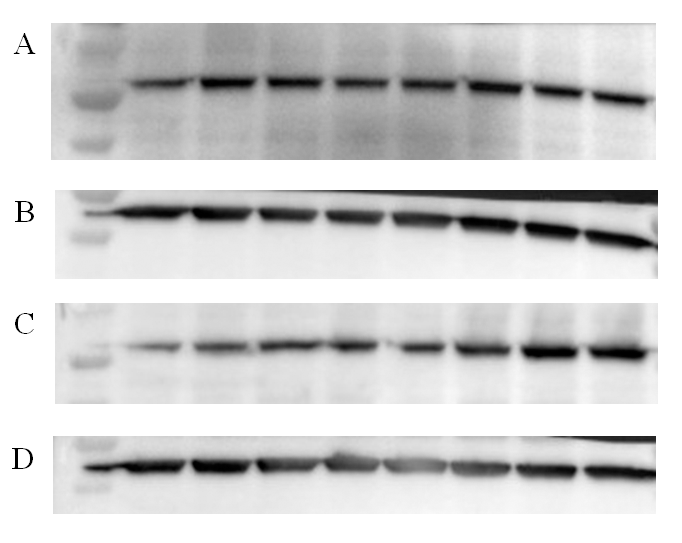

Supplement: Supplementary Fig. 1 [file srep34910-s2.tiff]

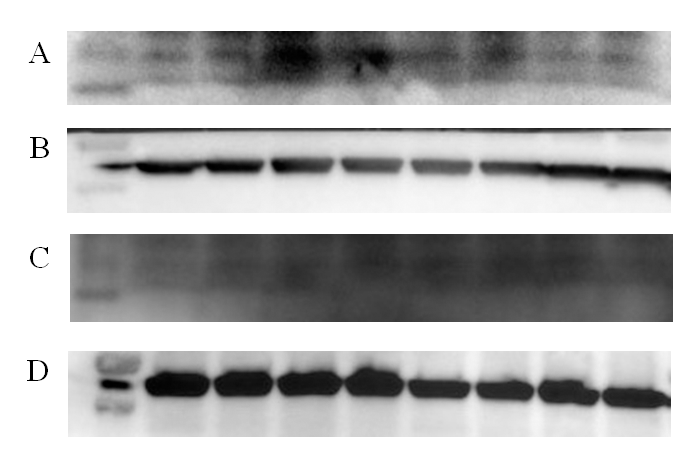

Supplement: Supplementary Fig. 2 [file srep34910-s3.tiff]
